# Comparative Effects of an Acute Bout of Self-Myofascial Release on the Plantar Fascia Using Auramat^®^ Versus Traditional Warm-Up on Quadriceps Function and Flexibility

**DOI:** 10.3390/healthcare14060757

**Published:** 2026-03-18

**Authors:** Danilo Gaias, Antonio Martínez-Serrano, Manuel Sanz-Matesanz, David Blanco-Luengo, Luis Manuel Martínez-Aranda

**Affiliations:** 1 UCAM Research Center for High Performance Sport, Catholic University of Murcia, 30107 Murcia, Spain; danilo.gaias26@gmail.com (D.G.); martinez.serrano97@gmail.com (A.M.-S.); 2 Strength and Conditioning Society, 30008 Murcia, Spain; 3 Faculty of Health Sciences, European University Miguel de Cervantes, 47012 Valladolid, Spain; msanzm@uemc.es; 4 Faculty of Sports Sciences, Department of Sports and Computer Sciences, Universidad Pablo de Olavide, 41089 Seville, Spain; dblalue@upo.es; 5 Physical Activity Analysis Research Group (SEJ-046), Department of Sport and Computer Science, Universidad Pablo de Olavide, 41089 Seville, Spain; 6 Centro Universitario San Isidoro, Universidad Pablo de Olavide, 41092 Seville, Spain; 7 Science-Based Training Research Group (SEJ-680), Physical Performance and Sports Research Center, Universidad Pablo de Olavide, 41089 Seville, Spain

**Keywords:** SMFR, knee extensors, force output, range of motion, superficial back line, time efficient

## Abstract

**Background**: Self-myofascial release (SMFR) is a treatment whose main benefits are enhanced recovery and increased flexibility without impairing athletic performance. Previous research has often targeted the posterior myofascial chain (superficial back line, SBL), which runs from the plantar fascia to the posterior cranium and is commonly linked to hamstring-related outcomes. However, its potential influence on knee extensor force production remains unclear and would likely be indirect. Many SMFR tools have entered the market in recent years, with Auramat^®^ being one of them, yet it has not been investigated to date. Therefore, this study aimed to determine the effects of Auramat^®^ (AUR) on posterior-chain flexibility and knee extensor (KE) function and to compare them with those of a traditional warm-up (TW). **Methods**: This study was a randomised, counterbalanced, cross-over design where 20 recreationally active participants (12 males, 8 females; age = 27.20 ± 4.98 years) attended the laboratory 3 times over a 2-week period. The first week consisted of a familiarisation session during which participants performed several tests. In the second week, the groups that were randomly assigned at AUR or TW conditions performed the two intervention protocols separated by 48 h. The pre-post ratings of perceived exertion (RPE), maximal voluntary isometric contraction (MVIC), straight leg raise test (SLRT) and rate of force development (RFD) were measured. All tests were performed on the dominant limb. **Results**: There was no significant difference in RFD and MVIC for conditions (*p* = 0.91), time (*p* = 0.24), or condition × time (*p* = 0.41). Both conditions improved posterior chain flexibility (*p* ≤ 0.01) with a larger effect in TW (*d* = 2.03; ↑ 7.81%) compared to the AUR condition (*d* = 0.89; ↑ 3.69%). RPE for TW showed significant higher RPE values compared to the AUR condition (*p* ≤ 0.01; ES = 2.32; TW = 4.3 ± 1.45 vs. AUR = 1.55 ± 0.82). **Conclusions**: Both SMFR with AUR and TW increased flexibility without any significant reduction in KE force production. Practitioners may use TW in a session where the aim is an increase in flexibility and AUR when the time is limited and the increase in fatigue can be relevant, due to the lower RPE reported. In any case, these results should be taken with caution since even the AUR was more time-efficient; the findings are preliminary owing to the small sample and absence of a control condition.

## 1. Introduction

Self-myofascial release (SMFR) has gained popularity among athletes and clinicians for its potential to enhance recovery and performance [1,2,3], with minimal contraindications or adverse effects [4]. It has been shown to reduce delayed onset muscle soreness (DOMS) [5], pain [5,6], improve arterial and vascular endothelial function [7,8], and modulate autonomic nervous system activity [9]. Despite widespread use in sport, the exact mechanisms remain unclear. Proposed explanations include both mechanical factors, such as thixotropy, fascial adhesions, and cellular fluid dynamics, and neurophysiological responses involving mechanoreceptors and the Golgi reflex arc [10].

Scientific attention has focused on the fascia’s role in transmitting tension. Myers et al. [11] mapped fascial connections, “anatomy trains”, suggesting that tension in one area may affect remote mobility. Consequently, many studies focus on the posterior chain, or superficial back line (SBL), which runs from the plantar fascia to the anterior cranium and is linked to hamstring injuries. Although the association between hamstring flexibility and injury risk is debated, recent approaches consider multifactorial interactions over simple cause–effect models [12]. Given the mechanical continuity of the SBL, alterations in tension or flexibility at the plantar fascia level may propagate proximally and potentially influence lower-limb neuromuscular function. In this context, changes in posterior-chain tension could indirectly affect knee joint mechanics and the functional behaviour of the quadriceps.

In this way, Franz et al. [13] demonstrated how hip flexor overactivity may induce anterior pelvic tilt and lumbar hyperlordosis, limiting hip extension and increasing hamstring strain. In this regard, Patel et al. [14] found that two minutes of SMFR on the plantar fascia using a tennis ball improved hamstring flexibility (*p* = 0.05; PREright = 20.46° ± 8.82°; POSTright = 27.46° ± 6.57°; PREleft = 21.80° ± 8.19°; POSTleft = 27.40° ± 4.83°). On the other hand, authors such as Do et al. [15] observed SBL flexibility gains in healthy adults following five minutes of plantar SMFR with a mini foam roller (rightPLSR↑ = 8.53° ± 4.74°; leftPLSR↑ = 8.46° ± 5.79°; TT↑ = 4.66 cm ± 2.66 cm). Grieve et al. [16] reported similar improvements in hamstring and lumbar flexibility after two minutes of tennis ball SMFR.

In general terms, available evidence indicates that self-myofascial release (SMFR) can be an effective strategy to improve joint range of motion (ROM) and flexibility in athletes. These benefits have been reported both when SMFR is applied in isolation (to a lesser extent) and, more consistently, when combined with stretching, particularly dynamic stretching, without impairing muscle activity or key performance outcomes such as strength, speed, and agility. In turn, enhanced ROM may facilitate more efficient movement patterns and potentially reduce the risk of skeletal muscle injuries [17].

Even though various tools such as foam rollers, sticks, or balls are used for SMFR, protocols remain inconsistent regarding duration, pressure, and tool choice. Despite this, it seems that reported application times range from ~30 s to 15 min, with a commonly used duration of approximately 1 min 30 s per muscle area [17].

**Figure 1:**
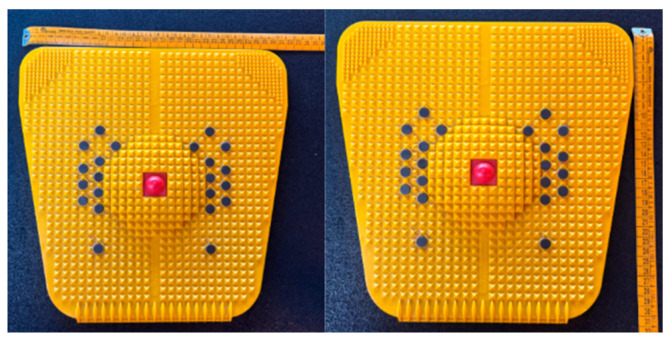
Auramat^®^ platform starter. Top length, 31 cm; bottom length, 31 cm. Centre point height, 2.5 cm. Spike height, 4 mm.

Unlike traditional SMFR tools such as foam rollers or tennis balls, which primarily apply compressive rolling forces over a larger surface area, the Auramat^®^ (~31 × 31 cm, Figure 1) uses a rigid platform covered with small spikes that deliver localised mechanical stimulation to the plantar surface. This structural configuration may lead to different patterns of pressure distribution and mechanoreceptor activation, potentially influencing fascial tension and neuromuscular responses along the superficial back line. It can be used while standing or during warm-up exercises performed above it. Brief plantar-surface SMR protocols have been shown to acutely increase posterior-chain flexibility even with short exposures (e.g., ≤2 min), and SMR is typically self-applied using body weight, which may help to standardise the relative pressure applied across participants [18,19]. SMFR may serve as a warm-up strategy to enhance flexibility without impairing force or power output [17].

As for traditional warm-ups, they typically combine aerobic exercise, mobility work, stretching, and sport-specific drills [20], with the aim of preparing the athlete for subsequent activity and reducing injury risk [21], ideally within the shortest effective time. In this context, the FIFA 11+ warm-up has been shown to reduce injury risk in team sports like football and can be completed in approximately 10–15 min [22]. In that regard, re-warm-up strategies appear effective in mitigating performance drops after stopping the activity, i.e., in team sports [23,24]. Russell et al. [25] suggested halftime re-warm-ups, which enhance sprint and jump ability [26,27]. Yet their effects on flexibility, an important determinant of both performance and injury prevention, have not been examined in depth. This is particularly relevant in sport contexts where halftime breaks may be limited to 2–6 min [28], highlighting the need for time-efficient strategies. The SMFR has gained popularity as a warm-up tool, but it remains unclear whether it can replace traditional methods. If effective, Auramat^®^ may offer a low-effort and time-efficient halftime intervention that could be applied passively in locker rooms while players rest and receive tactical instructions.

Interestingly, research on SMFR has mainly targeted the same muscle group treated, and no studies have assessed its effects on antagonist muscle force. The hamstring–quadriceps relationship is crucial for injury prevention [29,30,31], especially in ACL-related mechanisms [32], where receptor stimulation elicits protective hamstring responses [33]. Joint injuries may trigger flexor withdrawal reflex (FWR) facilitation, as seen in knee osteoarthritis [34,35] and ACL rupture cases with shortened biceps femoris latency [30]. Altered reflexes contribute to Arthrogenic Muscular Inhibition (AMI), impairing quadriceps strength and function [36,37].

Given SMFR’s potential to reduce muscle tension and alpha motor neuron excitability [9,10,38], plantar fascia SMFR could reduce hamstring tension, may raise FWR thresholds, and potentially benefit quadriceps force production; however, this remains to be tested.

Due to insufficient evidence supporting the interchangeability of SMFR and traditional warm-ups, and no prior studies that have examined the Auramat^®^, this research will intend to compare a ≥3–4 min Auramat^®^ plantar SMFR protocol with a standard 10–15 min dynamic warm-up. We hypothesised that Auramat^®^ would (i) improve straight leg raise ROM and (ii) maintain MVIC and RFD of the knee extensors, with lower perceived effort and reduced time, thus providing similar benefits to traditional warm-up.

## 2. Materials and Methods

### 2.1. Experimental Approach to the Problem

**Figure 2:**
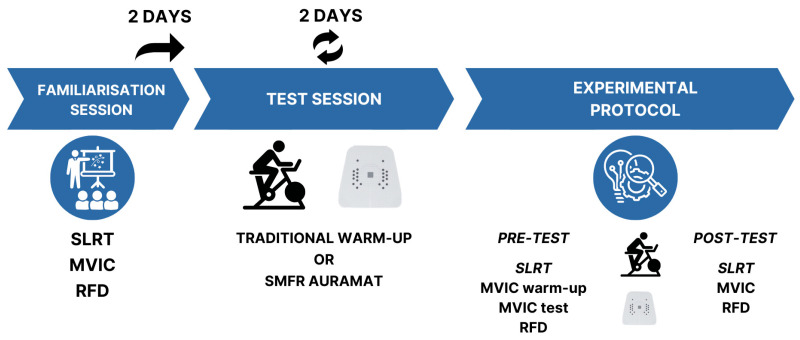
Study design. MVIC = maximal voluntary isometric contraction; SLRT = straight leg raise test; RFD = rate of force development.

This study employed a randomised, counterbalanced, cross-over design. Participants attended the laboratory three times over a two-week period, with the study design illustrated in Figure 2. Prior to the start of the study, participants were randomly assigned to the order in which they would perform each experimental condition using online software (Blia.it; https://www.blia.it/). Participants were given a number from 1 to 20 based on the order they applied for the study. Ten random numbers were selected using the software to perform the TW intervention first, while the remaining ten participants began with AUR. Participants were not informed about the intervention they would perform until their arrival at the laboratory.

The first experimental session involved a familiarisation phase, where all participants completed a test consisting of the following: three trials of the straight leg raise test; maximal voluntary isometric contraction (MVIC) of the knee extensors, with three warm-up repetitions of 5 s each, followed by 45 s of rest between repetitions; after 2 min, three full-effort contractions of 5 s each, with 2 min of rest between each repetition. After another 2 min, the rate of force development (RFD) of the knee extensors was measured, with five repetitions of 1–2 s each, resting 20 s between repetitions. The familiarisation session and each intervention session were separated by at least two days.

During the second and third sessions, participants performed the two different interventions, ensuring that both groups underwent both protocols or conditions across two separated sessions. A single researcher supervised all sessions and collected the measurements to ensure procedural consistency across participants. Due to the nature of the interventions and the study logistics, assessor blinding was not implemented. The SLRT → MVIC → RFD sequence was identical across sessions, leaving a complete recovery between tests in order to avoid fatigue carry-over (eccentric hamstring loading during SLRT; possible post-activation changes due to RFD collected after MVIC).

### 2.2. Subjects

Twenty recreationally active participants (12 males, 8 females; age = 27.20 ± 4.98 years; weight = 76.40 ± 12.14 kg; height = 1.73 ± 0.09 m) voluntarily participated in this study. Participants were instructed to avoid strenuous exercise for 48 h prior to testing and to maintain their usual diet and fluid intake throughout the study. Subjects were excluded from the study if they

- Had knee pain or injury (within the last 6 months).
- Were taking anti-inflammatory medication or additional supplementation daily during the study period.

After reading the information sheet, participants provided informed consent. The study procedure was approved by the local Ethics Committee (code: CE072312), in accordance with the Helsinki Declaration.

### 2.3. Procedures

#### 2.3.1. Straight Leg Raise Test for Posterior Chain Flexibility

**Figure 3:**
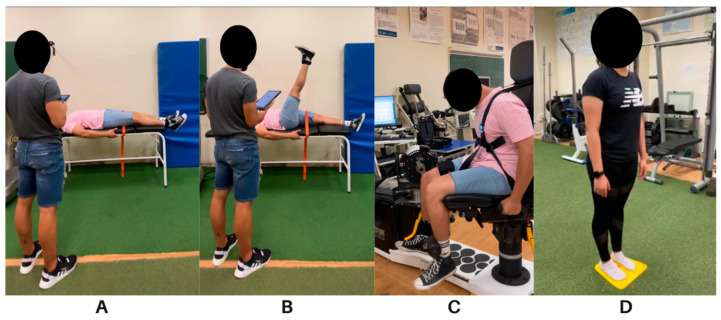
Methods. (**A**,**B**) Starting and final position during measurements for straight leg raise test. (**C**) Participant’s position during MVIC and RFD measurement. (**D**) Foot placement during Auramat^®^ intervention.

Participants were positioned supine on a physiotherapy table and asked to raise their dominant leg as much as possible, holding it for two seconds at the highest point, while keeping the leg straight and the ankle at a 90° angle throughout the test (Figure 3A,B). A portable inertial sensor with a goniometer (K-move/KFORCE, Kinvent^TM^, Montpellier, France) was placed inside a pocket in an ankle strap to measure the maximum range of motion.

Although this technology is relatively new in sport settings, previous studies using the same Kinvent electrogoniometer (Kivent Ibérica, Barcelona, Spain) platform have reported excellent inter-rater and test–retest reliability for lower-limb range-of-motion (ROM) measurements (ICC = 0.86–0.98), together with strong concurrent validity versus universal goniometry (r > 0.89). Additionally, low measurement error has been reported for ROM assessment (SEM ≈ 0.18–0.52), supporting its use for joint ROM quantification [39].

The contralateral leg was strapped at the thigh to the physiotherapy table to prevent compensation. Three repetitions were performed, and the maximum value was recorded for analysis. The trial was considered invalid if the tested leg was not kept straight during the test, the ankle was not bent at 90°, or the contralateral thigh was lifted off the table.

#### 2.3.2. Knee Extensor Maximal Voluntary Isometric Contraction

Participants performed the MVIC test using an isokinetic dynamometer (Humac Norm, Computer Sports Medicine, Stoughton, MA, USA). The test was conducted with participants seated on a chair, with their knee bent at 90°. Restraints were applied around the thigh and trunk, and the ankle was placed in a padded strap attached to a strain gauge (Figure 3C). Before beginning the test, participants completed a warm-up consisting of three submaximal MVICs at 65%, 85%, and 95% of perceived exertion, with 45 s of rest between repetitions. Following a two-minute rest, participants completed the test, which involved three full-effort repetitions of five seconds each, with two minutes of rest between repetitions. Prior to each MVIC, participants were instructed to perform the contraction “as hard as possible” [40] and were motivated throughout the test with verbal encouragement. The test was interrupted and repeated if the straps became dislodged or the knee angle changed.

#### 2.3.3. Knee Extensor Rate of Force Development (RFD)

The RFD test was performed using the same isokinetic dynamometer as the MVIC (Humac Norm, Computer Sports Medicine, Stoughton, MA, USA). Force data were acquired using Trigno Discover software ver. 2.1. (Delsys Incorporated, Natick, MA, USA) at a frequency of 1 kHz. RFD was measured two minutes after the last MVIC. Participants maintained the same position as during the MVIC test, but the ankle was wrapped in the stiffer part of the strap to reduce compliance during the measurement. They were asked to perform five repetitions of 1–2 s each, with 20 s of rest between repetitions. All participants were instructed to perform the contraction “as fast and hard as possible” [40] and were encouraged throughout the effort with verbal motivation. The contractions were visually inspected by the researcher, and those with a countermovement were immediately discarded, as was the case for the MVIC test. The three best repetitions, which exhibited good execution and the highest peak force, were used for analysis. The average of the best three repetitions for TW and AUR in both pre- and post-measurements were considered for the analysis.

### 2.4. Interventions

The traditional warm-up (TW) consisted of ~8 min of cycling (Technogym, Cesena, Italy) at Level 4 with RPM between 50 and 60, where heart rate was monitored. After 1′ rest, two sets of twelve repetitions of dynamic stretching (standing hip abduction/adduction and standing hip flexion/extension) were performed with no rest between sets. The warm-up concluded with two sets of ten repetitions of bodyweight squats and walking lunges with 1′ minute between sets and exercise. Dynamic stretching, squat and walking lunges pacing were standardised for all the subjects. The total duration of the intervention was ~14 min.

The Auramat^®^ intervention involved standing on the platform (Platform “Starter” Auramat, Jodhpur, India) without shoes (wearing standard socks) and for ~3–4 min while gently moving the centre of mass forward and backward without losing balance (Figure 3D). The duration of the Auramat^®^ intervention (~3 min) was selected based on previous studies showing that short plantar SMFR exposures (e.g., 2–5 min) can produce measurable improvements in posterior-chain flexibility [15,16]. The time between pre- and post-measures during the Auramat^®^ intervention was at least five–six minutes.

After three to five minutes following each intervention, participants were asked to rate their perceived exertion (RPE) during the interventions using the Borg CR-10 Scale [41,42].

### 2.5. Data Analysis

For both the straight leg raise test (SLRT) and maximal voluntary isometric contraction (MVIC), the best repetition out of three was used for statistical analysis. Rate of force development (RFD) torque analysis was performed using LabChart 8 (ADinstruments-Dunedin, New Zealand). First, the reliability of the contractions was verified by discarding those with an unstable baseline or countermovement. The peak force for each repetition was then detected, and the best three repetitions were selected for analysis. For each of the three repetitions, the onset of the contraction was visually defined following a specific criterion: “the last trough before force deflects above the range of the baseline noise” [43]. A marker was placed at the onset of the contraction, and force values were recorded at 50 ms, 100 ms, 150 ms, and 200 ms from the onset. For each variable, the average of the best three repetitions was taken for statistical analysis.

### 2.6. Sample Size and Power Analysis

A sensitivity power analysis was conducted using G*Power (v3.1) to determine the smallest effect size detectable with the available sample. Given the randomised, counterbalanced crossover design, the primary effect of interest (condition × time interaction) can be expressed as a within-participant comparison of pre–post change scores between conditions (ΔTW vs. ΔAUR), equivalent to a paired-samples framework. With n = 20, α = 0.05 (two-tailed), this study had 80% power to detect a standardised effect size of dz = 0.66 and 90% power to detect dz = 0.76. Therefore, the present sample was adequate to detect moderate-to-large acute effects, whereas smaller effects (e.g., dz = 0.50) would be expected to have lower power (≈56%). In line with recommendations to consider effect-size uncertainty and replicability in sports and exercise science, effect sizes are reported alongside *p*-values [44].

### 2.7. Statistical Analysis

Test–retest reliability of SLRT, MVIC, and RFD was assessed using intraclass correlation coefficients (ICC) with 95% confidence intervals, applying a two-way mixed-effects model with absolute agreement. Given the data reduction approach, ICC was computed according to the final score used in the analyses: for SLRT and MVIC, where the best repetition out of three was retained, reliability was expressed as ICC (3,1) (single measurement; best-of-three). For RFD, where the outcome was defined as the average of the best three repetitions, reliability was expressed as ICC (3,3) (average measures). Normality of the residuals was inspected using the Shapiro–Wilk test. For the main outcomes (SLRT, MVIC, and RFD at 50 ms, 100 ms, 150 ms, and 200 ms), a two-way repeated-measures ANOVA (2 × 2) was conducted in accordance with the randomised, counterbalanced crossover design, with condition (TW vs. AUR) and time (pre vs. post) as within-subject factors. When applicable, sphericity was evaluated using Mauchly’s test, and the Greenhouse–Geisser correction was applied if the assumption was violated. If significant main effects or interactions were detected, post hoc pairwise comparisons were performed with adjustment for multiple comparisons. Ratings of perceived exertion (RPE) were compared between conditions using a paired-samples *t*-test. Statistical significance was set at *p* ≤ 0.05. Effect sizes were calculated as Cohen’s *d* [45] (within-subject/paired) using an online calculator (socscistatistics.com). The magnitude of Cohen’s *d* (regardless of sign) was interpreted as very small (0.01), small (0.20), medium (0.50), large (0.80), very large (1.20), and huge (2.0), as originally proposed by Cohen [46] and expanded by Sawilowsky [47].

## 3. Results

The results for each of the variables analysed are as follows.

### 3.1. Flexibility

A significant time effect (*p* < 0.01) was found, with a post hoc analysis showing a mean difference of 4.66° between pre- and post-measurements. The test revealed that flexibility increased independently of the experimental group. Although no significant effects were observed in either condition (*p* = 0.67) or in the condition × time interaction (*p* = 0.23), the change in TW was slightly higher (*d* = 2.03; ↑ 7.81%; *p* < 0.01) than in AUR (Table 1). Intra-session flexibility measurements showed excellent reliability (ICC = 0.99, CV < 5%).

### 3.2. MVIC

**Figure 4:**
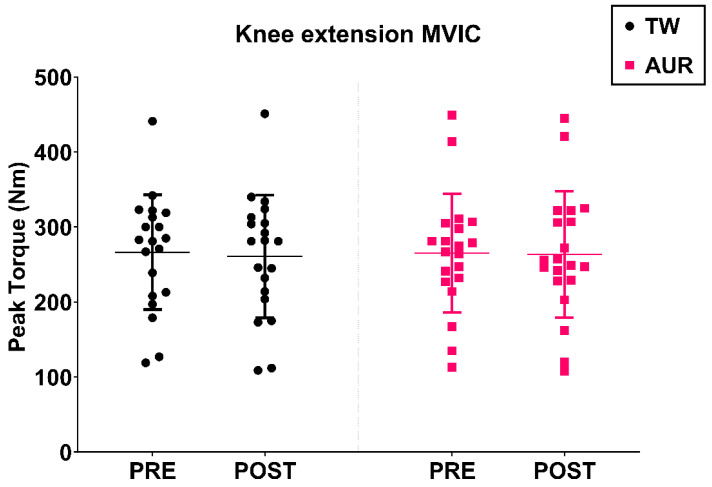
Knee extension MVIC pre–post-measurements.

No significant effects were observed in the two-way repeated-measures ANOVA for condition (*p* = 0.91), time (*p* = 0.24), or the condition × time interaction (*p* = 0.41). (Figure 4). Intra-session MVIC reliability measurements were excellent (ICC = 0.99, CV < 5%).

### 3.3. RFD

No significant effects were found with a two-way ANOVA in all the factors analysed (condition/time/condition × time) for RFD at 50 ms,100 ms,150 ms and 200 ms (Table 2). RFD measurements were reliably performed. RFD100 ms, 150 ms and 200 ms showed excellent reliability (ICC > 0.90) while RFD50 ms showed moderate reliability (ICC = 0.59).

### 3.4. RPE

We found a significant difference in RPE between AUR and TW interventions, being the RPE of AUR significantly lower than TW (*p* ≤ 0.01; *d* = 2.32; AUR = 1.55 ± 0.82 vs. TW = 4.30 ± 1.45).

## 4. Discussion

### 4.1. Main Findings

This study sought to examine the acute effects of Auramat^®^ (AUR) on posterior-chain flexibility and knee extensor (KE) function and to compare these responses with those elicited by a traditional warm-up (TW). The main findings were as follows: (i) Both interventions significantly improved posterior-chain flexibility over time; however, no statistically significant differences were found between conditions. Although the descriptive effect size was larger after TW (*d* = 2.03; +7.81%) compared with AUR (*d* = 0.89; +3.69%), the repeated-measures ANOVA did not reveal a significant condition or condition × time interaction, indicating that the interventions cannot be statistically distinguished based on the present data. (ii) Neither intervention produced significant changes in KE force production, as reflected by MVIC and RFD assessed at 50, 100, 150, and 200 ms; and (iii) perceived exertion was substantially higher during TW compared with AUR. Taken together, AUR appears to provide a modest but measurable acute flexibility stimulus with lower perceived exertion, whereas TW showed slightly greater descriptive flexibility gains, although no statistically significant differences were observed between conditions.

### 4.2. Interpretation and Comparison with Previous Studies

The flexibility improvements observed after AUR align with previous SMFR studies targeting the plantar fascia, which have reported significant increases in hamstring- and lumbar-related flexibility outcomes [14,15,16,48]. However, important methodological differences should be considered. Prior studies commonly applied plantar-fascia SMFR in seated positions using tennis balls, lacrosse balls, or foam rollers, with pressure titrated to discomfort (without pain). In contrast, participants in the present study stood on a rigid platform while shifting their centre of mass forwards and backwards, with the relative pressure largely determined by body mass. Although this approach may have contributed to some degree of standardisation across participants, the actual plantar pressure was not quantified and may still have varied between individuals.

Regarding neuromuscular performance, direct comparisons are limited because plantar-fascia SMFR studies have seldom included KE function outcomes. Nonetheless, our null findings for MVIC and RFD are consistent with work showing that brief SMFR/foam rolling can increase ROM without immediately impairing force production. For example, MacDonald et al. [49] reported increased quadriceps ROM after a short foam-rolling bout with no significant immediate changes in RFD or MVIC. Similarly, no MVIC changes were found after rolling massage applied for 3 min to the hamstrings under constant pressure (13 kg for 5–10 s), despite significant ROM improvements in the sit and reach test [50]. In contrast, a small increase in MVIC (4%), apart from ankle ROM, was reported after 90 s roller massage of the calf muscle, although the authors attributed this to a likely rise in muscle temperature rather than a direct neuromuscular potentiation effect [51]. It is also worth noting that delayed effects may occur: MacGregor et al. [52] found no immediate MVIC changes after quadriceps foam rolling like in our study but reported a significant increase 30 min later. Meanwhile, another study by MacDonald [5] found that foam rolling helped alleviate muscle soreness while improving vertical jump height, muscle activation, and both passive and dynamic ROM after exercise-induced muscle damage. However, their results cannot be directly compared with ours as they were part of a chronic study with repeated measures taken at 0, 24, and 48 h post-exercise. Therefore, the absence of changes in MVIC or RFD may be partly explained by the immediate timing of post-intervention assessments, and future studies should consider multiple post-intervention time points.

Finally, our findings should not be interpreted as evidence that AUR and TW are interchangeable. Although both interventions improved flexibility and neither reduced KE force output, formal equivalence testing was not performed. Therefore, conclusions regarding functional equivalence should be made cautiously and within the exploratory nature of the present design.

### 4.3. Potential Mechanisms

The acute flexibility gains following plantar-fascia SMFR are likely driven by non-structural mechanisms, particularly given the short intervention duration and the absence of detectable changes in KE MVIC and RFD. A plausible explanation may involve changes in stretch tolerance or sensory modulation, whereby plantar stimulation could alter the perception of stretch discomfort and allow greater ROM without necessarily altering muscle force-generating capacity in the knee extensors. Such effects may be related to stimulation of cutaneous and fascial mechanoreceptors located in the plantar surface, which have been associated with neurosensory modulation and reflex responses influencing neuromuscular behaviour [9,10]. Under the present conditions, any neuromuscular changes induced by plantar stimulation were not large enough to translate into measurable differences in maximal or explosive KE output.

With respect to RFD specifically, we analysed discrete time windows (50, 100, 150, and 200 ms) and found no significant effects at any time point. Early RFD measures (e.g., 50 ms) are known to be more variable and more dependent on neural factors than later phases, which may reduce sensitivity to small intervention effects [53]. However, the very small observed effect sizes (e.g., d ≈ 0.14) suggest that any practical influence of either AUR or TW on explosive KE force production was minimal under the tested conditions. Because none of the RFD comparisons approached significance, the risk of Type I error inflation due to multiple testing is acknowledged but unlikely to affect interpretation; similarly, applying an alpha correction method such as Holm–Bonferroni would not alter the interpretation of the results. Nonetheless, future studies with larger samples and near-threshold effects may benefit from a family-wise correction approach or a linear mixed model framework to simultaneously account for the temporal dimension and interindividual variability.

### 4.4. Limitations and Future Directions

Several limitations should be considered when interpreting these findings. First, while the crossover design and randomisation help to mitigate some concerns, the absence of a true control condition and the lack of assessor blinding limits causal inference. This design cannot fully rule out potential practice effects, measurement variability, or other uncontrolled influences. Second, the time between pre- and post-testing differed between interventions (AUR > 3–4 min vs. TW ~14 min), which may have affected outcomes, particularly flexibility, and complicated direct comparisons. Third, measurements of MVIC and RFD were obtained immediately after the interventions; as suggested by delayed improvements reported elsewhere [52], additional post-tests at later time points (e.g., 10–30 min) may be necessary to capture time-dependent neuromuscular responses. Fourth, the stimulation intensity delivered by the Auramat^®^ may have been attenuated by slight differences in socks, potentially reducing the plantar stimulus and increasing interindividual variability.

A further limitation relates to the sample and external validity. The participants were recreationally active rather than athletes, and responses to recovery or warm-up strategies can differ between trained and untrained populations [54]. This should be considered when extrapolating to competitive settings. Future studies should include a control condition, quantify plantar pressure (to verify standardisation), standardise footwear/socks, incorporate athlete samples, and include multiple post-intervention time points. Larger samples would also allow more definitive evaluation of small effects and enable equivalence or non-inferiority approaches if interchangeability is a key applied question.

In addition, Cohen’s *d* was used as the primary effect-size metric due to its interpretability across within- and between-condition contrasts. The magnitude thresholds were interpreted following Cohen and the extended classification proposed by Sawilowsky [45,46,47]. While partial η^2^ is commonly reported in ANOVA contexts, standardised mean differences were prioritised here to facilitate practical interpretation of the observed changes.

### 4.5. Practical Applications

From an applied perspective, TW produced slightly greater acute flexibility gains, whereas AUR produced smaller improvements with substantially lower perceived exertion. This trade-off may be relevant in contexts where time is limited and minimising effort is desirable. A practical scenario is the half-time break in football, where performance can decline during the initial period of the second half compared with the first half [23], being applicable to other sport disciplines or contexts. A complete passive break may not be optimal, and re-warm-up strategies have been proposed to attenuate these declines [24,25] and can improve sprint and jump performance [26,27]. Although flexibility outcomes have not been directly studied within half-time re-warm-up research, flexibility is considered a relevant factor for football performance and has been linked to hamstring injury risk [55]. Moreover, reductions in flexibility may occur across match play; for instance, decreases in adductor flexibility have been reported as match duration increases [56]. Because practitioners report having only 2–6 min available for re-warm-up during half-time [28], a brief and low-effort strategy such as Auramat^®^ may represent a practically attractive option. This tool could provide a flexibility stimulus without compromising immediate KE force production. Nevertheless, the practical relevance of a ~3.7% flexibility gain remains uncertain, and sport-specific trials are required to determine whether these acute changes translate into meaningful performance or injury-related outcomes.

Beyond sport performance settings, a plantar-focused SMFR stimulus may also be relevant in rehabilitation contexts. In patients with plantar fasciitis, a randomised controlled trial reported that a brief foam-roller intervention produced acute improvements in pain and ankle range of motion [57]. This supports the idea that a short plantar-focused SMFR stimulus could be used as an adjunct within rehabilitation warm-ups when clinicians want mobility gains without adding high exertion. Therefore, although the present findings were obtained in recreationally active participants and cannot be directly extrapolated to clinical populations, Auramat^®^ could be explored as a time-efficient option for delivering plantar stimulation when minimising exertion is desirable.

## 5. Conclusions

In conclusion, in line with our hypothesis, this study demonstrated that both self-myofascial release (SMFR) using Auramat^®^ and a traditional warm-up improved posterior chain flexibility over time without detrimental effects on knee extensor force production. However, no statistically significant differences were detected between interventions, despite the larger descriptive effect size observed for the traditional warm-up. Given its brief duration and lower RPE values, the Auramat^®^ intervention appears to offer a time-efficient alternative, although further research is needed to confirm its equivalence with traditional warm-up protocols and to explore the mechanisms involved.

## Figures and Tables

**Table 1:** Descriptive data of SLRT and MVIC tests shown as mean ± SD, % change pre–post and Cohen’s *d*.

	Pre	Post	Mean Diff.	C.I (95%)	%Change	Cohen’s *d*
SLRT_TW (°)	80.19 ± 3.17	86.46 ± 2.99	6.27	[2.04, 10.49]	7.81	2.03
SLRT_AUR (°)	82.45 ± 3.22	85.50 ± 3.58	3.05	[0.53, 6.63]	3.69	0.89
MVIC_TW (N·m)	266.45 ± 17.10	260.85 ± 18.32	−5.60	[−2.32, 13.52]	−2.14	0.31
MVIC_AUR (N·m)	265.35 ± 17.70	263.35 ± 18.82	−2.00	[−6.15, 10.15]	−0.69	0.01

Note: AUR = Auramat^®^; MVIC = maximal voluntary isometric contraction; SLRT = straight leg raise test; TW = traditional warm-up.

**Table 2:** Descriptive data of RFD measurements shown as mean ± SD, % change pre–post and Cohen’s *d*.

	Pre	Post	Mean Diff.	C.I (95%)	%Change	Cohen’s *d*
RFD50_TW (N·m)	292.02 ± 127.27	316.15 ± 220.51	24.13	[−85.71, 37.45]	8.26	0.12
RFD50_AUR (N·m)	248.43 ± 224.06	325.54 ± 253.06	41.11	[−55.5, 137.7]	31.03	0.33
RFD100_TW (N·m)	729.56 ± 332.69	709.79 ± 389.31	−19.77	[−82.85, 122.39]	−2.78	0.05
RFD100_AUR (N·m)	733.18 ± 354.57	725.46 ± 278.59	−7.71	[−87.22, 102.65]	−1.06	0.02
RFD150_TW (N·m)	692.90 ± 267.37	743.10 ± 279.71	50.19	[41.95, 142.33]	7.24	0.18
RFD150_AUR (N·m)	694.16 ± 257.15	732.13 ± 265.74	37.97	[31.53, 107.48]	5.46	0.14
RFD200_TW (N·m)	635.29 ± 255.80	598.91 ± 243.61	−36.37	[−42.43, 115.19]	−6.07	0.14
RFD200_AUR (N·m)	631.45 ± 239.46	663.19 ± 229.16	31.74	[−68.1, 131.5]	5.02	0.13

Note: AUR = Auramat^®^; RFD = rate of force development; TW = traditional warm-up.

## Data Availability

The raw data supporting the conclusions of this article will be made available by the corresponding or last authors of the manuscript on request.
